# Prognostic implication and functional exploration for microRNA-20a as a molecular biomarker of gastrointestinal cancer

**DOI:** 10.1186/s12885-020-06875-5

**Published:** 2020-05-14

**Authors:** Qiliang Peng, Peifeng Zhao, Yi Shen, Ming Cheng, Yongyou Wu, Yaqun Zhu

**Affiliations:** 1 Department of Radiotherapy & Oncology, The Second Affiliated Hospital of Soochow University, Suzhou, China; 2 Institute of Radiotherapy & Oncology, Soochow University, Suzhou, China; 3 Department of Radiation Oncology, The Affiliated Suzhou Science & Technology Town Hospital of Nanjing Medical University, Suzhou, China; 4 Department of General Surgery, The Second Affiliated Hospital of Soochow University, Suzhou, China

**Keywords:** Gastrointestinal cancer, microRNA-20a, Prognosis prediction, Function exploration

## Abstract

**Background:**

It is generally accepted that microRNA-20a (miR-20a) is aberrantly expressed in gastrointestinal cancer (GIC), and may be associated with the prognosis of GIC patients. Nevertheless, the clinical prognostic value of miR-20a expression in GIC remains controversial.

**Methods:**

We first conducted a comprehensive literature search of the clinical data and pooled them for evidence in assessing prognostic significance of miR-20a expression in GIC. Afterwards, we applied some bioinformatic analysis methods to explore the biological function of miR-20a and explain why miR-20a could act as an effective biomarker.

**Results:**

The pooled results showed that enhanced miR-20a expression was significantly associated with poor survival in GIC patients (HR: 1.36; 95%CI: 1.21–1.52; *P* < 0.001). According to the subgroup analysis, the ethnicity, cancer type, sample source, and sample size may have an impact on the predictive roles for miR-20a. The gene ontologies enriched by the predicted miR-20a targets were highly associated with some important biological processes, cell components and molecular functions. Moreover, a series of prominent pathways linked with GIC carcinogenesis were identified. Ultimately, the crucial targets and modules were identified by constructing the protein-protein interaction network of miR-20a targets, which were highly associated with the initiation and progression of GIC according to previous molecular biology experiments.

**Conclusions:**

Our results indicated that high expression of miR-20a may be a credible indicator of worse prognosis in GIC. Further studies involving biological experiments and larger sample sizes should be performed to validate these findings.

## Background

Gastrointestinal cancer (GIC), one of the most common malignancies, has overtaken cardiovascular disease and infectious diseases as a significant health burden with the leading cause of mortality across the world because of the growing incidence each year and poor prognosis [1]. Although diagnostic and therapeutic strategies for GICs have been greatly improved, the prognosis of these patients remains very unsatisfying according to the latest statistics [2]. Currently, TNM stage-based predictive system and some markers such as CEA play important roles in the monitoring and prognosis of GIC. However, there is still no effective biological biomarkers to understand the cancer development and tumor behavior and promote more precise risk stratification, as well as optimal choice of therapy [3]. Hence, it is urgently needed to explore new credible prognostic markers which could be applied to supplement the current TNM stage-based predictive system and to provide guidance for cancer therapy.

The microRNAs are small single-stranded RNA molecules that mediate the downstream gene expression in a post-transcriptional manner [4]. An increasing number of recent studies have emphasized the roles of microRNAs in a variety of biological activities such as proliferation, apoptosis, angiogenesis, invasion, and migration [5]. Due to its stability and detectability in tissues and blood, microRNAs might function as promising biomarkers for cancer early diagnosis, prognosis or treatment responses prediction [6].

Notably, miR-20a stands out as the most investigated example in functional microRNAs. Recently published work has implicated its significant function in cancer pathogenesis and during the initiation and progression processes of carcinogenesis [7]. Furthermore, accumulating new evidence demonstrates that aberrant expression of miR-20a may be highly associated with initiation and metastasis in GIC [8]. Nevertheless, there are inconsistencies regarding the prognostic value of miR-20a in GIC, though a large number of studies reported associations between miR-20a expression and the clinical outcomes [9].

Thus, through a comprehensive literature search of the relevant studies, we conducted an integrated meta-analysis regarding the influence of miR-20a expression level on overall survival of GIC patients. Additionally, functional exploration by bioinformatic analysis was performed to provide a better understanding of the prognostic significance for miR-20a involved in the occurrence and development of GIC, aiming to provide more theoretical supports for targeted treatment.

## Methods

### Literature retrieval strategy

Two researchers (QP and PZ) independently conducted a systematic computerized literature search for available studies in selected electronic databases of PubMed, EMBASE and Web of science until October 2019. Search keywords were (microRNA-20a OR miR-20a OR miR20a OR miRNA-20a OR miRNA20a) AND (colorectal OR colon OR rectal OR rectum OR gastric OR gastrointestinal OR stomach) AND (tumor OR neoplasm OR cancer OR carcinoma OR malignancy). We also retrieved studies by hands from other potentially qualified publications to complement the results including relevant meta-analyses, reviews and references cited in these papers.

### Inclusion criteria and exclusion criteria

All the studies were included if they met the following inclusion criteria: (1) Studies concentrated on pathological diagnosed GIC patients; (2) The associations between miR-20a expression and the survival of GIC patients were described; (3) The hazard ratios (HRs) and their corresponding 95% confidence interval (CIs) for overall survival based on miR-20a expression either had to be directly provided or could be estimated from the information presented.

Studies were removed if they met any of the following criteria: (1) Literatures such as conference records, abstracts, reviews or meta-analysis; (2) Studies without enough data to obtain trustworthy HRs and corresponding 95% CIs; (3) Articles were published in languages other than English.

### Data extraction and quality assessment

The following information was collected from each eligible study: first author; year of publication; patients characteristics (age; ethnicity; country); specimen type; technical methodology; sample size; follow-up times; prognostic parameters (HRs and 95%CIs). If the HRs and 95%CIs were not directly given by the original research, they were extracted from the Kaplan-Meier curves with the methods stated by Tierney et al. [10]. Newcastle-Ottawa Scale (NOS) was applied to appraise the methodological quality of enrolled studies [11]. Generally, study with more than 6 score indicated a high quality. Two authors (QP and PZ) separately performed these procedures, after which a cross-check was accomplished and disagreements were discussed with a third reviewer to reach consensus.

### Data synthesis methods

We combined the HRs and the 95% CIs to quantitatively evaluate the influence of miR-20a expression on the prognosis of GIC patients. The random-effects model was applied to obtain the pooled HRs if significant heterogeneity was determined by the I^2^ metric (I^2^ ≥ 50%) and Cochran Q test (*P* ≤ 0.10) [12]. If no obvious heterogeneity was observed, a fixed-effect model would be utilized for further analysis. Additionally, we also explored potential variables of heterogeneity through subgroup analysis and meta-regression analysis [13]. Meanwhile, to evaluate the sources of heterogeneity, we further conducted sensitivity analysis. At last, the publication bias was assessed by Begg’s test and Egger’s test [14]. In our study, all above statistical were accomplished using STATA version 12.0 software. *P*-value < 0.05 was deemed as statistically significant.

### Identification of target genes

The targets of miR-20a were predicted using miRTarBase, which is experimentally validated microRNA-target interaction database. In the most recent edition, this database contained > 13,404 validated microRNA-target interactions collected from 11,021 articles based on manual collection and integration [15].

### Functional annotation by KEGG and GO analysis

To analyze the biological function annotation information of miR-20a targets, an integrative characterization of miR-20a targets were explored. Gene ontology (GO) is a tool designed for annotating genes, collecting and analyzing information based on cellular component (CC), biological process (BP) and molecular function (MF) levels [16]. Kyoto encyclopedia of genes and genomes (KEGG) database is an online analysis tool to integrate and interpret large molecular datasets [17]. To perform GO and KEGG analysis of miR-20a targets, the Database for Annotation, Visualization and Integrated Discovery (DAVID version 6.8) online tool was applied [18]. *P* < 0.05 was considered statistically significant.

### PPI network construction and network module analysis

Search Tool for the Retrieval of Interacting Genes (STRING), an online open database, collects comprehensive data on proteins to evaluate the protein-protein interaction (PPI) information [19]. We selected STRING database to obtain the PPI data among miR-20a targets. Interactions with a Combined Score of > 0.4 were collected and then visualized with Cytoscape software [20]. Subsequently, the CytoNCA plug-in was used to identify hub genes according to three different centrality measures, including betweenness centrality and closeness centrality and degree centrality [21]. In addition, the MCODE plug-in of Cytoscape, was applied to identify the critical modules of the network map. Ultimately, the KEGG pathway analysis was chosen to explore the involvement of the hub nodes and module nodes in different biological pathways.

## Results

### Literature search

**Fig. 1:**
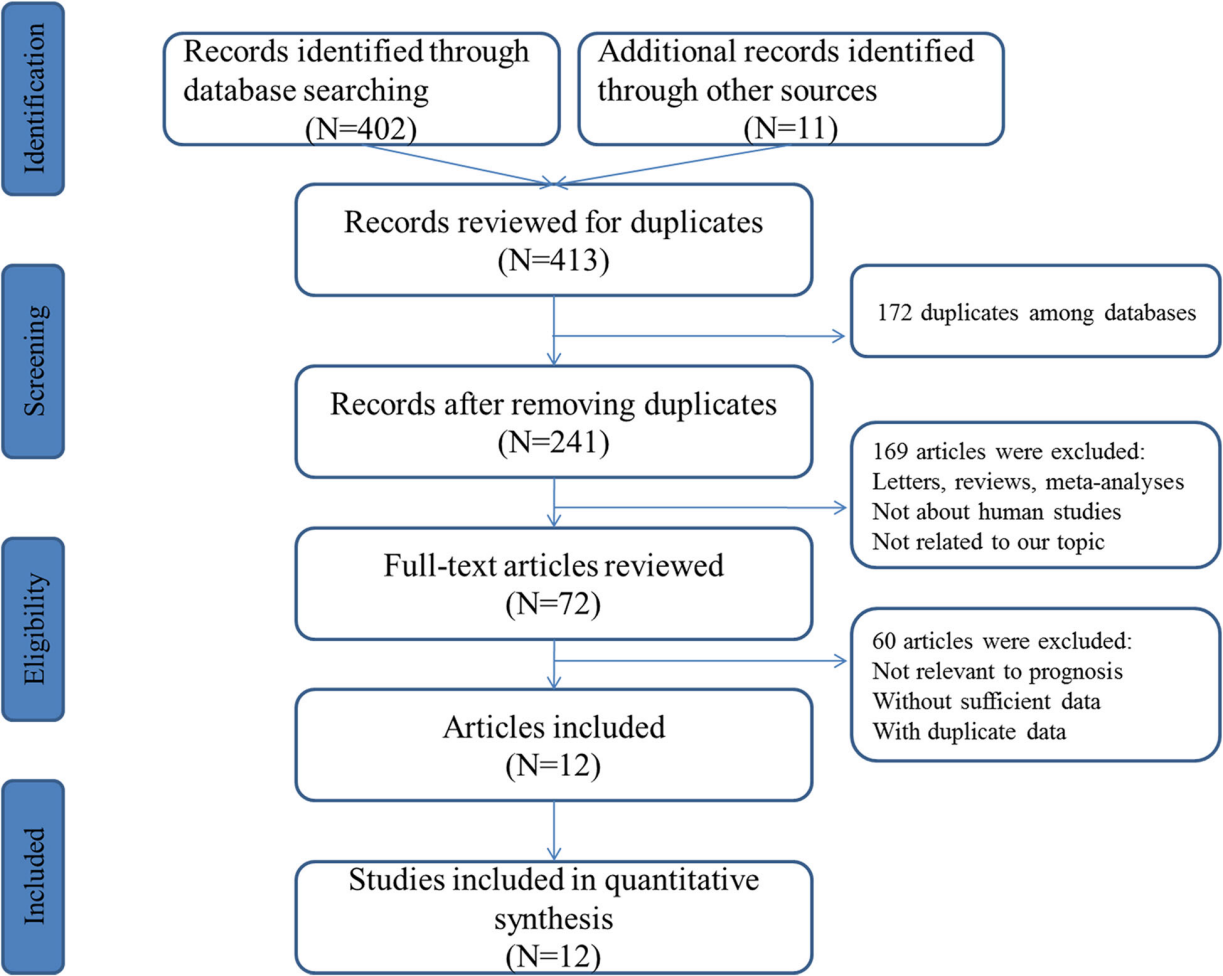
Flow diagram of filtering studies

According to the criteria, a search conducted on PubMed, Web of Science and EMBASE originally identified 402 relevant publications. In addition, 11 potentially relevant citations were obtained through manually scanning the references of these articles. After the exclusion of duplicate literatures, 241 publications were then retained. Nevertheless, 229 records were removed after reading the titles, abstracts or full texts. Ultimately, we enrolled 12 articles including 12 studies for data pooling [22–33]. Figure 1 exhibited the flow chart used for literature search.

### Characteristics of the included studies

The characteristics of the studies enrolled for data pooling were summarized in Table 1. Briefly, 12 studies were included, which were published between 2008 and 2019. The total number of participants included in the present study was 1927. These studies were conducted in Asian (*n* = 9) and Non-Asian populations (*n* = 3). There were seven studies on gastric cancer (GC), four studies on colorectal cancer (CRC) and one study on GIC (contained gastric cancer and colorectal cancer). The sample sources were classified as tissue (*n* = 7) and blood (*n* = 5). All the studies measured miR-20a by reverse-transcription quantitative polymerase chain reaction (RT-qPCR).

**Table 1:** Characteristics of the included articles

Author	Year	Country	Ethnicity	M/F	N	Age	Cancer type	TNM stage	Sample source	Methods	Endpoints	Median follow-up time	Hazard ratio
Schetter et al	2008	USA	Non-Asians	66/18	84	65	CRC	I-IV	Tissue	RT-PCR	OS	68	2.20 (1.10–4.60)
Ayerbes et al	2011	Spain	Non-Asians	25/13	38	63	GIC	I-IV	Tissue	RT-PCR	OS	22	1.07 (1.00–1.13)
Osawa et al	2011	Japan	Asians	26/11	37	65	GC	I-IV	Tissue	RT-PCR	OS	38	1.20 (1.12–1.58)
Kim et al	2012	Korea	Asians	57/34	91	61	GC	I-IV	Tissue	RT-PCR	OS	46	1.19 (0.83–1.69)
Wang et al	2012	China	Asians	43/22	65	60	GC	I-IV	Blood	RT-PCR	OS	36	1.58 (1.10–2.25)
Huang et al	2014	China	Asians	52/30	82	60	GC	I-IV	Blood	RT-PCR	OS	20	1.08 (1.02–1.15)
Chen et al	2015	China	Asians	NR	580	NR	CRC	I-IV	Tissue	RT-PCR	OS	NR	1.88 (1.09–3.23)
Cheng et al	2016	China	Asians	264/280	544	65	CRC	I-IV	Tissue	RT-PCR	OS	110	8.22 (4.47–15.12)
Yang et al	2017	China	Asians	35/20	55	60	GC	I-IV	Blood	RT-PCR	OS	34	2.30 (1.60–3.32)
Peng et al	2018	China	Asians	179/154	333	59	GC	I-III	Blood	RT-PCR	OS	36	2.07 (1.36–3.15)
Shao et al	2018	China	Asians	NR	NR	NR	GC	NR	Tissue	RT-PCR	OS	NR	1.02 (1.01–1.03)
Pesta et al.	2019	Czech	Non-Asians	18/10	28	NR	CRC	I-IV	Blood	RT-PCR	OS	36	1.67 (1.07–2.60)

***Abbreviation***: *F* Female, *M* Male, *N* Number, *NR* Not report, *CRC* Colorectal cancer, *GC* Gastric cancer, *GIC* Gastrointestinal cancer, *OS* Overall survival

### Pooled prognostic value of miR-20a in gastrointestinal cancer

**Fig. 2:**
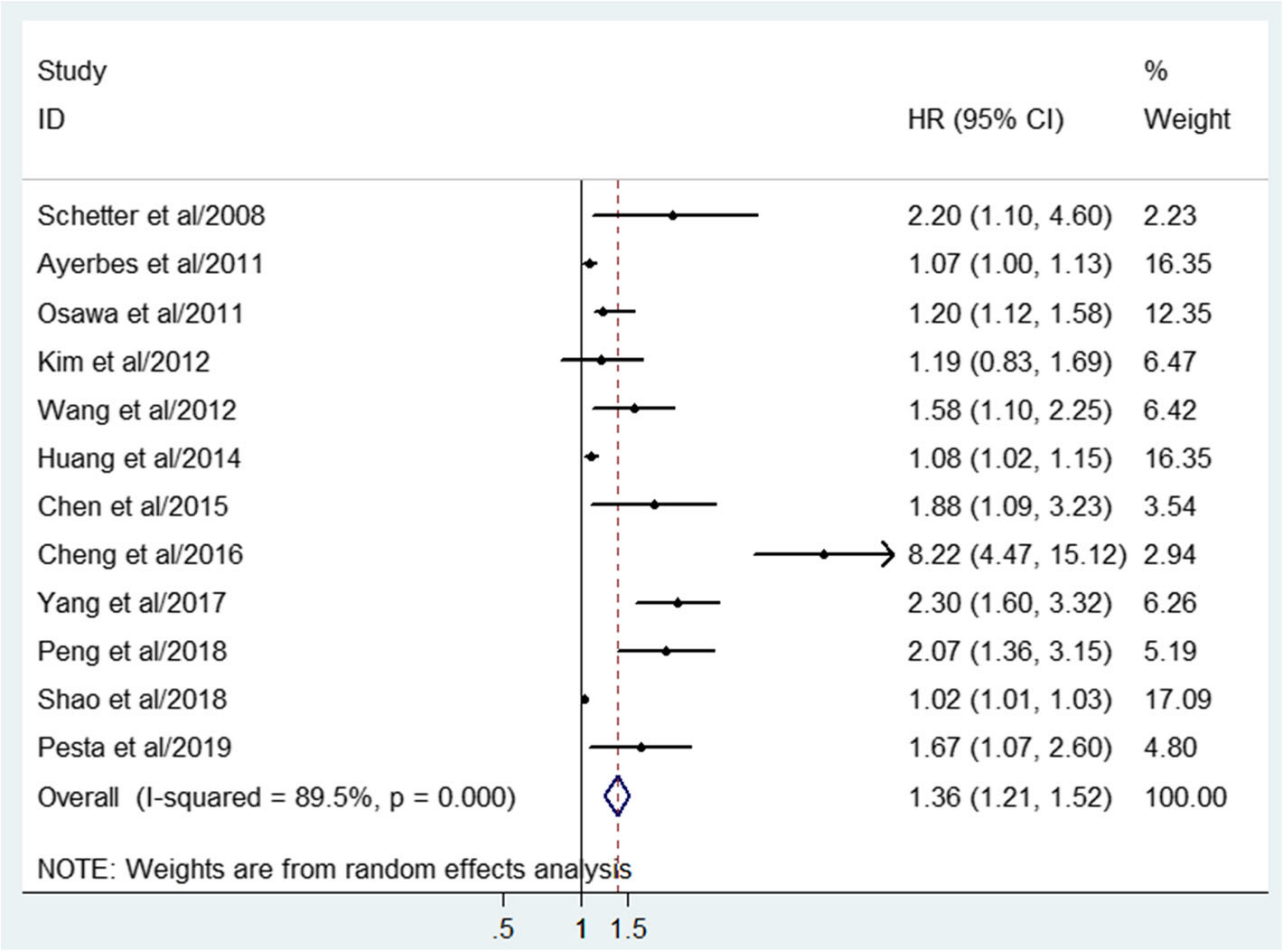
Forest plot of the relationship between miR-20a and overall survival in GIC. GIC, gastrointestinal cancer

A random-effect model was applied to generate the combined association between miR-20a expression level and overall survival of GIC patients, since highly significant heterogeneity (I^2^ = 89.5%, *P* < 0.001) was detected when twelve studies were pooled (Fig. 2). The pooled analysis indicated that up-regulated miR-20a expression was significantly linked with worse OS in patients with GIC (HR: 1.36; 95%CI: 1.21–1.52; *P* < 0.001).

### Subgroup analysis and meta-regression analysis

To explore the sources of heterogeneity, subgroup analysis was performed according to the main characteristics (Table 2). Subgroup analysis by ethnicity explored that up-regulated miR-20a expression status was identified to be a worse prognostic biomarker in Asians group (HR: 1.46; 95%CI: 1.25–1.71; *P* < 0.001), but not in non-Asians group (HR: 1.43; 95%CI: 0.92–2.23; *P* = 0.11). Afterwards, the results revealed that the predictive role of miR-20a was significant in both blood sample (HR: 1.65; 95%CI: 1.14–2.37; *P* = 0.008) and tissue sample (HR: 1.29; 95%CI: 1.11–1.50; *P* = 0.001). In addition, cancer type subgrouping indicated obvious associations between high expression of the miR-20a and poor OS in both GC (HR: 1.25; 95%CI: 1.10–1.40; *P* = 0.006), and CRC (HR: 2.71; 95%CI: 1.33–5.54; *P* < 0.001). Furthermore, large sample size revealed more significant predictive role than small sample size with a HR of 2.37 (95%CI: 1.29–4.33; *P* = 0.005) versus that of 1.25 (95%CI: 1.10–1.43; *P* = 0.001).

**Table 2:** Results of subgroup and meta-regression analyses

Subgroup	Studies	HR (95%CI)	***P***-value	Heterogeneity (I^**2**^)	P_**heterogeneity**_	Meta-regression (***P***-value)
**Ethnicity**						*P* = 0.776
Asian	9	1.46 (1.25–1.71)	*P* < 0.001	91.5%	*P* < 0.001	
Non-Asian	3	1.43 (0.92–2.23)	*P* = 0.11	74.1%	*P* = 0.021	
**Cancer type**						*P* = 0.189
Gastric cancer	7	1.25 (1.10–1.40)	*P* = 0.006	86.4%	*P* < 0.001	
Colorectal cancer	4	2.71 (1.33–5.54)	*P* < 0.001	84.2%	*P* < 0.001	
**Sample source**						*P* = 0.851
Blood	5	1.65 (1.14–2.37)	*P* = 0.008	87.1%	*P* < 0.001	
Tissue	7	1.29 (1.11–1.50)	*P* = 0.001	90.2%	*P* < 0.001	
**Sample size**						*P* = 0.271
Large(>median)	5	2.37 (1.29–4.33)	*P* = 0.005	86.2%	*P* < 0.001	
Small(<median)	6	1.25 (1.10–1.43)	*P* = 0.001	80.4%	*P* < 0.001	

We also tried to apply the meta-regression analysis by considering some key variables to explore the prognostic role of miR-20a, such as ethnicity, cancer types, sample sources and sample sizes. Nevertheless, no clinical significance has been found (*P* > 0.05).

### Sensitivity analysis and publication bias

**Fig. 3:**
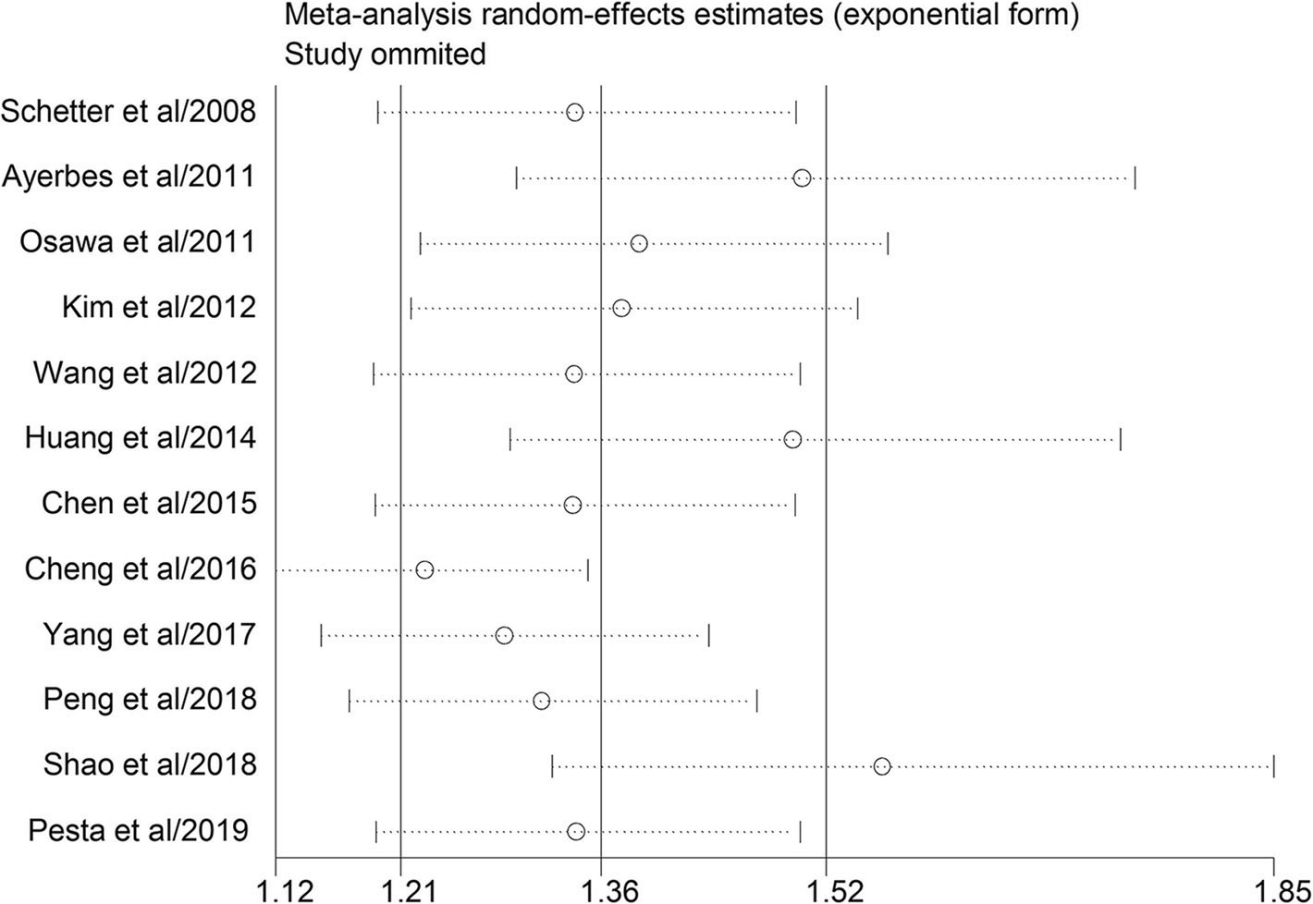
Sensitivity analysis for the pooled hazard ratios of overall survival of patients with high level of miR-20a expression. The sensitivity analysis was conducted to evaluate the stability of the pooled HR for OS by omitting one study at each step

Sensitivity analysis was then performed to test the robustness of the synthesized results of the effect of miR-20a on OS. We sequentially eliminated single study, and found that no single study significantly could cause heterogeneity (Fig. 3). Ultimately, potential publication bias across the enrolled prognostic studies was assessed by applying Begg’s funnel plot and Egger’s test. As a result, potential publication bias was detected in the included studies (*P* < 0.05).

### Functional characterization of miR-20a targets

**Fig. 4:**
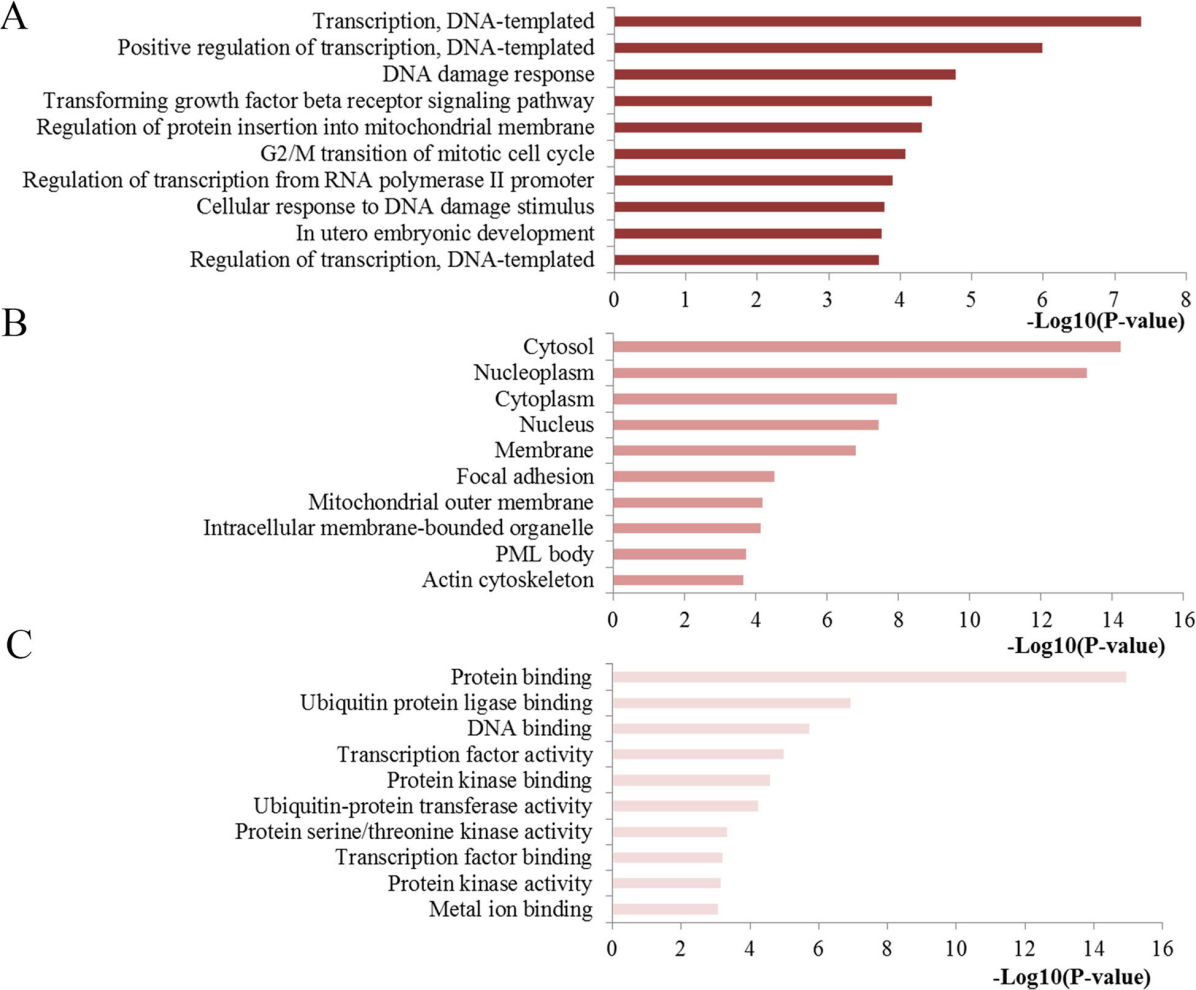
Top ten GO annotation results of miR-20a targets. **a** Biological processes (BP); **b** cell component (CC); **c** molecular function (MF). GO, gene ontology

The miR-20a targets were collected from miRTarBase. To understand whether the main biological function of miR-20a is associated with GIC, functional enrichment analysis of the miR-20a targets was performed by using the DAVID online tool. With respect to BPs, the target genes of miR-20a were mainly enriched in processes such as transcription, DNA damage response, transforming growth factor beta receptor signaling pathway and cell cycle. With respect to CCs, the target genes of miR-20a were mostly related to key cell component including cytosol, nucleoplasm, cytoplasm and nucleus. With respect to MFs, the target genes of miR-20a were highly linked with binding abilities such as protein binding, ubiquitin protein ligase binding, and protein kinase binding (Fig. 4).

**Fig. 5:**
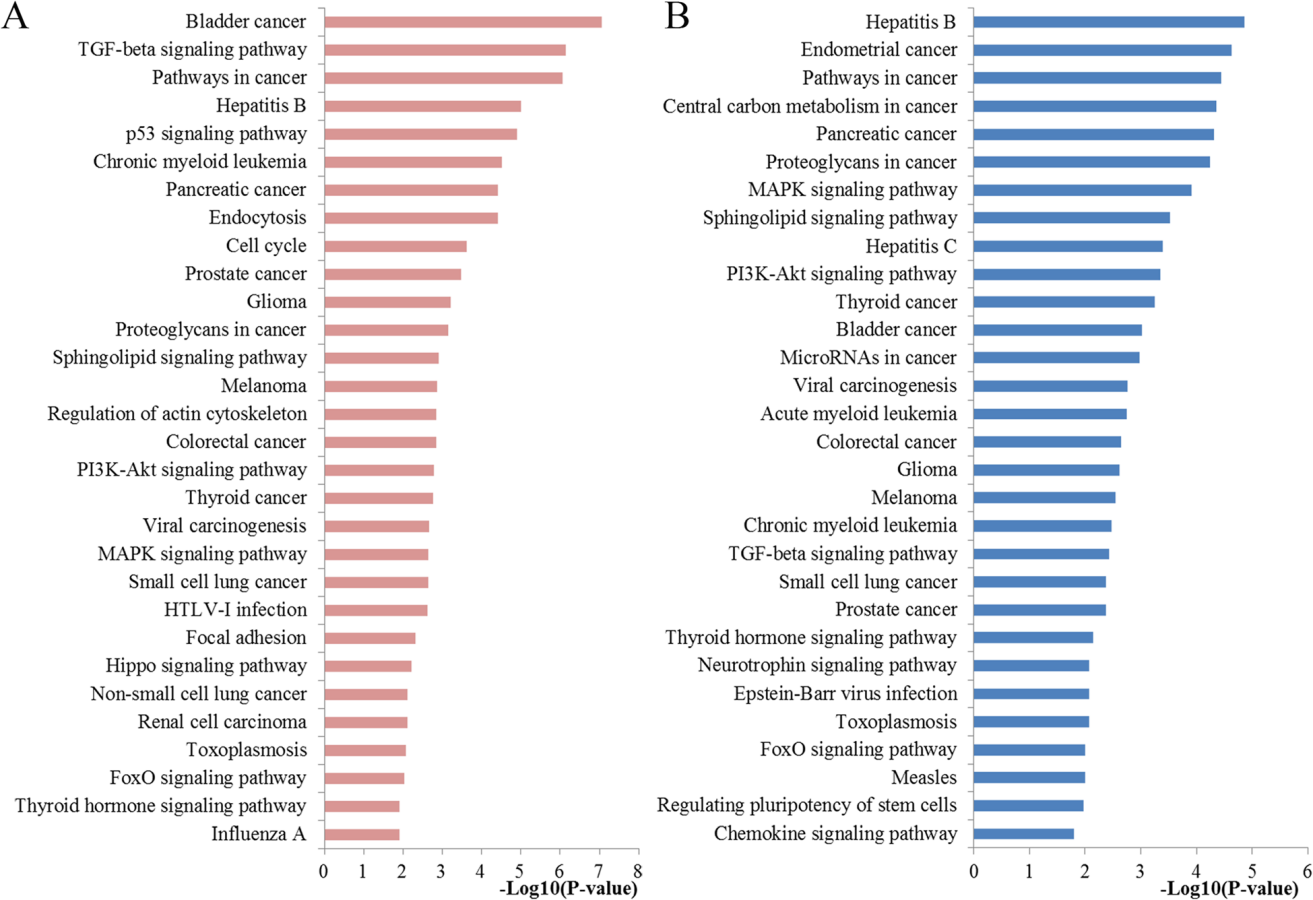
Pathway enrichment results. **a** Top 30 pathways enriched by all the targets of miR-20a; **b** Top 30 pathways enriched by the hub nodes of miR-20a. The Database for Annotation, Visualization and Integrated Discovery (DAVID version 6.8) online tool was applied to perform the pathway enrichment analysis

**Fig. 6:**
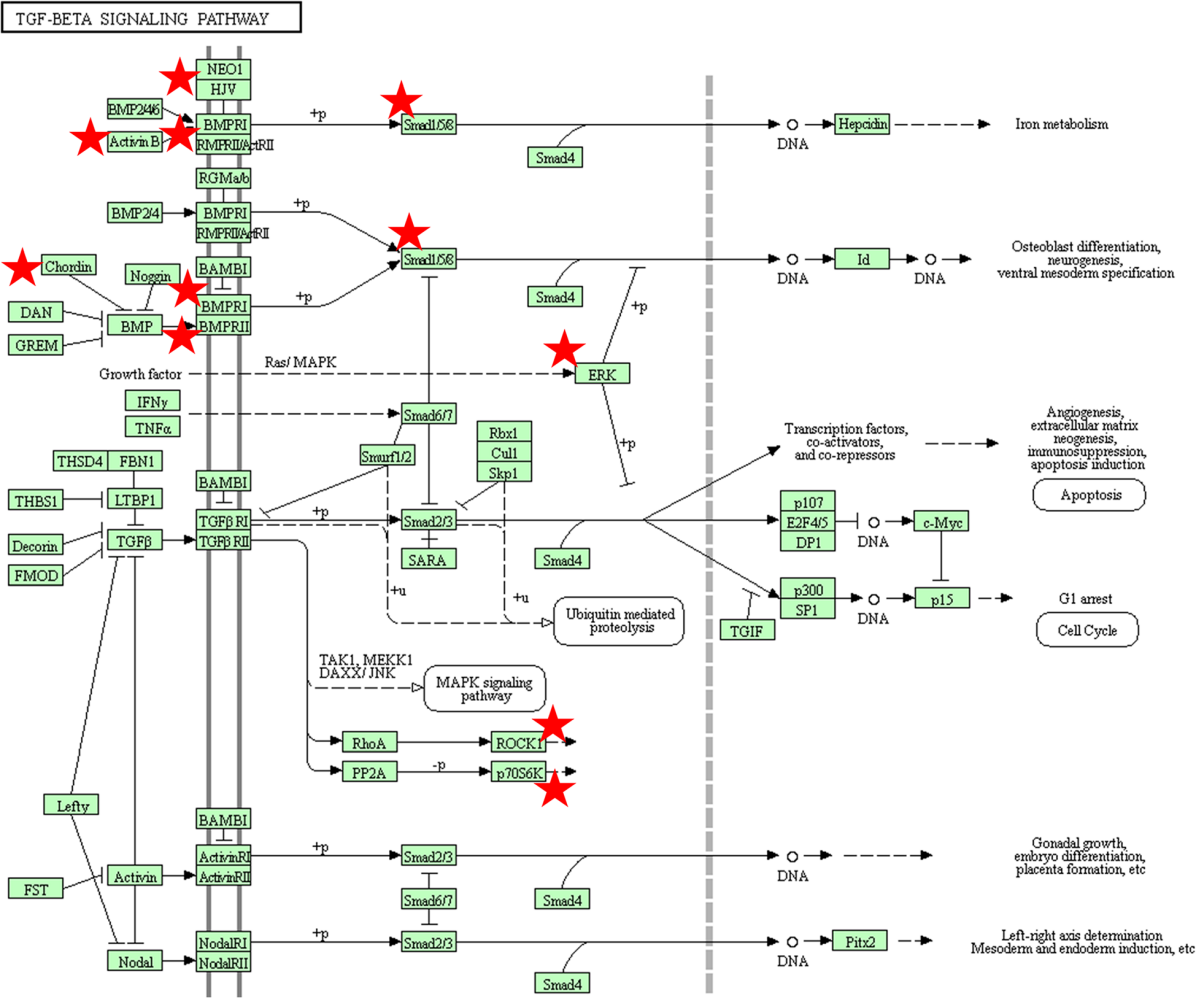
The TGF-beta signaling pathway enriched in KEGG. Objects with pentagrams are acting locus by mapped genes. TGF-beta, Transforming growth factor-beta; KEGG, Kyoto encyclopedia of genes and genomes

Subsequently, the results of KEGG pathway analysis revealed that the target genes of miR-20a were highly enriched in TGF-beta signaling pathway, pathways in cancer, p53 signaling pathway, cell cycle, Proteoglycans in cancer, sphingolipid signaling pathway, colorectal cancer, PI3K-Akt signaling pathway, viral carcinogenesis and MAPK signaling pathway. Figure 5 illustrated the top 30 enriched KEGG pathways. The most significant TGF-beta signaling pathway identified from KEGG was plotted at Fig. 6, which also has close connections with cell cycle, apoptosis and MAPK signaling.

### PPI network construction and hub gene selection

**Fig. 7:**
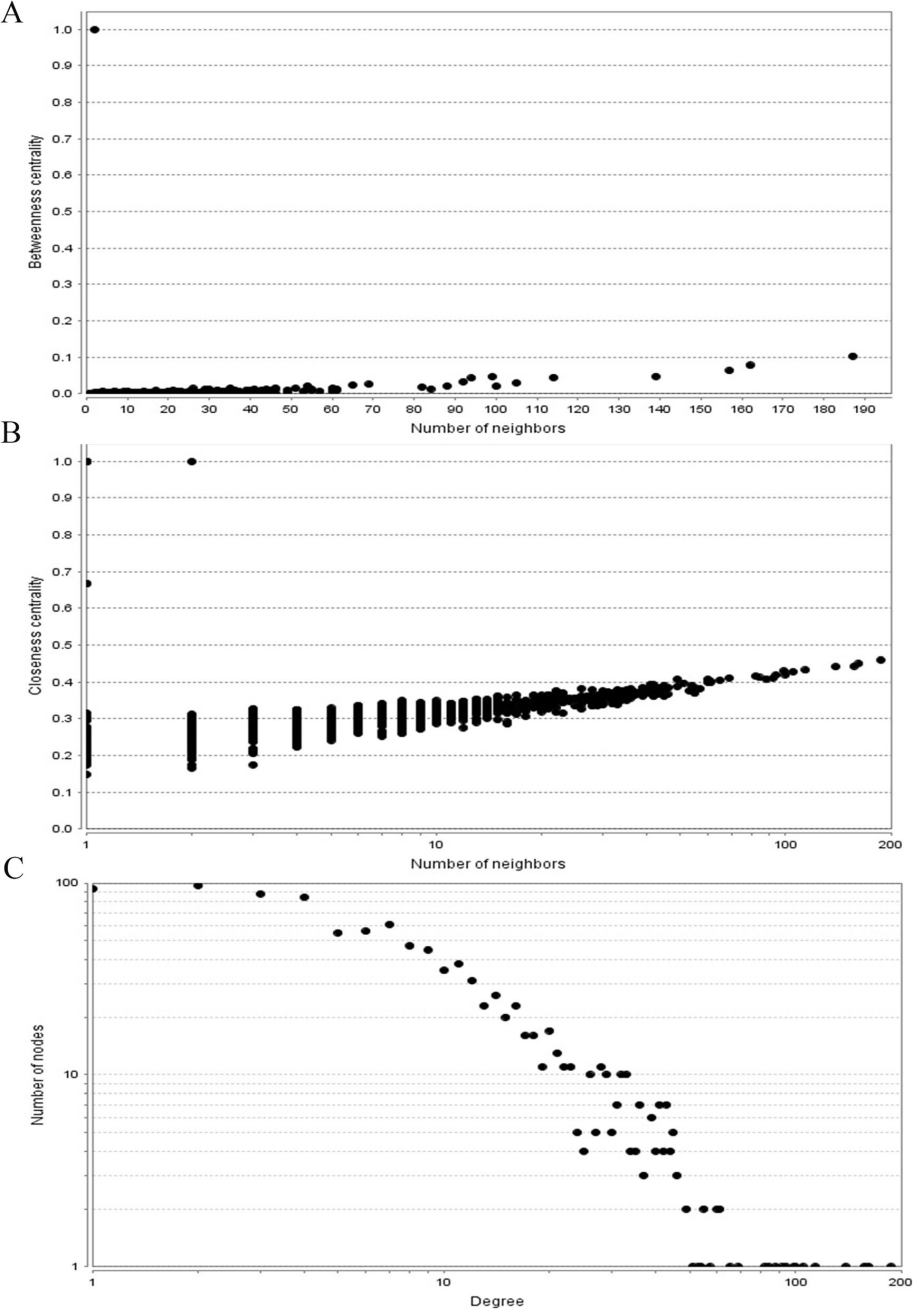
PPI network construction results. **a** Betweenness centrality distributions of nodes; **b** Closeness centrality distributions of nodes; **c** Degree distributions of nodes. PPI, protein-protein interaction

To predict the interactions between miR-20a targets at the protein level, a PPI network was set up using the STRING database. The PPI network of the miR-20a targets was set up consisting of 1019 nodes and 12.895 average numbers of neighbors. The network was then visualized with Cytoscape software for evaluating the interactions between the target genes of miR-20a in GIC. The CytoNCA plug-in of Cytoscape was employed for vital hub nodes from the PPI network through identifying the top ten nodes ranked by betweenness centrality, closeness centrality and degree centrality (Fig. 7). Subsequently, the top ten hub genes were identified including *TP53*, *UBC*, *RPS27A*, *MYC*, *HSPA8*, *MAPK1*, *CDC42*, *STAT3*, *PTEN*, and *PPP2R1A*. Functional analysis of KEGG pathways presented that hub genes were mainly enriched in several important signaling pathway such as pathways in cancer, central carbon metabolism in cancer, proteoglycans in cancer, MAPK signaling pathway, sphingolipid signaling pathway, PI3K-Akt signaling pathway, microRNAs in cancer, colorectal cancer, TGF-beta signaling pathway and FoxO signaling pathway.

### Identification of core modules and analysis of their function

**Fig. 8:**
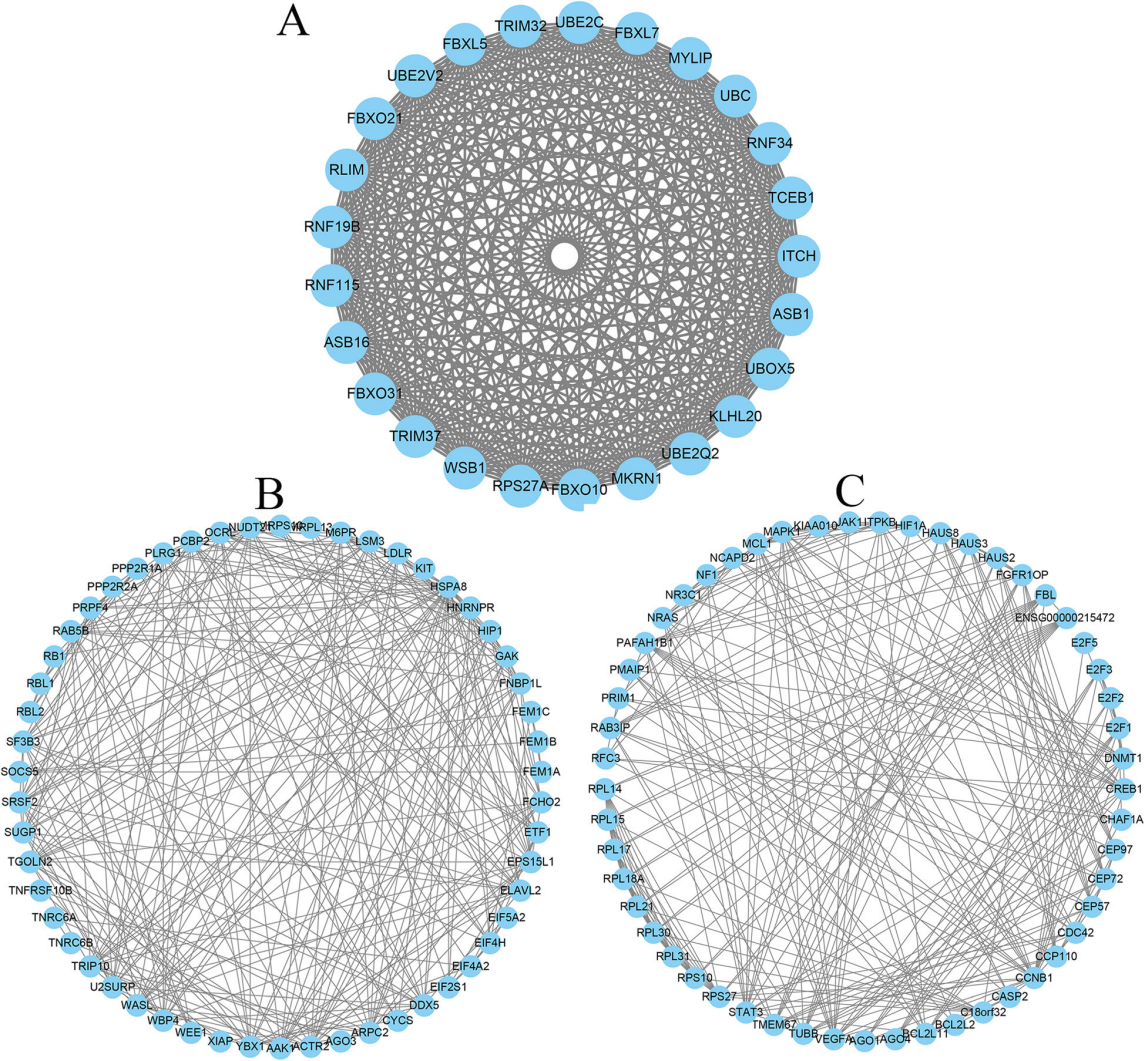
The top three significant modules of the PPI network. The three modules were identified and reconstructed with Cytoscape. PPI, protein-protein interaction

We used the MCODE plug-in to extract the significant modules of the PPI network with a score > 10 (Fig. 8), and then performed functional pathway enrichment analysis. The KEGG pathway analysis suggested that genes involved in the key modules were mostly enriched in ubiquitin mediated proteolysis, spliceosome, Endocytosis, mRNA surveillance pathway, microRNAs in cancer, Pathways in cancer, proteoglycans in cancer, cell cycle, VEGF signaling pathway, FoxO signaling pathway, PI3K-Akt signaling pathway, HIF-1 signaling pathway and Ras signaling pathway.

## Discussion

Numerous studies have been conducted to clarify the associations between miR-20a and the clinical outcomes of GIC, but the results to date remain inconclusive. Hence, it was deemed essential to perform a literature search of the relevant studies and carry out a meta-analysis of this issue. Furthermore, the occurrence and progression of GIC are complex and heterogeneous, with multiple cumulative genetic alterations, ultimately resulting in an aggressive condition. Consequently, there is also a great need to explore the molecular mechanisms for miR-20a involved in GIC.

We first performed a comprehensive meta-analysis to quantitatively synthesize the evidence pertaining to miR-20a as a predictive biomarker for patients’ prognosis by analyzing published studies concerning GIC. In this study, the pooled results revealed that the GIC patients with higher miR-20a expression had significantly worse OS than those with low miR-20a expression with the pooled HR of 1.36 (95%CI: 1.21–1.52; *P* < 0.001). Given that the promising results may be overshadowed by the significant heterogeneity (I^2^ = 89.5%, *P* < 0.001), we applied the random-effect model to generate the statistic parameters. In addition, several common methods were applied to seek the potential source of heterogeneity. According to the subgroup analysis, ethnicity may contribute to the prognosis difference for miR-20a as Asians with higher miR-20a expression were related to worse prognosis than that of Non-Asians. In addition, the subgroup analysis of sample type for miR-20a indicated that the predictive role of miR-20a was both significant in blood and tissue while high expression of miR-20a in tissue sample was associated with more unfavorable patients’ survival. Moreover, it was demonstrated from the results that miR-20a could be served as a useful biomarker for both GC and CRC. Interestingly, we also found that prognostic value of miR-20a was more remarkable in large-sample-size groups compared with small ones, indicating that more large-scales researches are required to decipher the prognostic value of miR-20a for GIC. But there are still a few deficiencies as potential publication bias was detected in the current study. Then meta-regression and sensitivity analysis were performed explore the impact of single clinical variable or single study on the predictive role of miR-20a. No significant results were found, suggesting the robustness of our study to some extent. In preliminary summary, the present study suggested that high miR-20a expression may function as an unfavorable indicator and intimately associated with deteriorated OS for patients with GIC.

We then applied an integrated bioinformatic analyses to explore the potential mechanism of miR-20a in GIC. To understand the potential function of miR-20a, the GO annotation and KEGG pathway were analyzed with the target genes. The results of the GO analysis in the present study indicated that miR-20a targets linked with BP were mostly enriched in a series of important processes including transcription, DNA damage response, TGF-beta receptor signaling pathway and cell cycle. Targets of miR-20a linked with CC were highly involved in key intracellular and extracellular spaces while regarding MF, miR-20a targets were significantly linked with key molecules binding. In addition, KEGG analysis indicated that miR-20a targets were enriched in several important signaling pathways. These enriched pathways have been validated by previous experimental investigations. In detail, Pathways in cancer contained various important signaling pathways, which directly influenced the progression of GIC. Colorectal cancer pathway demonstrated that miR-20a was really related to the occurrence and development of this disease [34]. TGF-beta signaling has been one of the most significant cellular pathways with pivotal roles in modulating cell growth, differentiation, apoptosis, and homeostasis in development of colorectal cancer [35, 36]. The well-studied p53 signaling has been implicated in extensive aspects of cellular activities, such as apoptosis, cell cycle arrest, senescence, metabolism, differentiation and angiogenesis [37]. The cell cycle signaling has been verified to be the hallmark of cancer that associated with cellular proliferation, the aberrant activation of which may result in uncontrolled cell proliferation, making them attractive therapeutic targets in cancer treatment [38]. Proteoglycans have been well established as key regulators in extensive normal and pathological processes, such as morphogenesis, tissue repair, inflammation, vascularization and cancer metastasis [39]. Studies have convinced the roles of sphingolipid signaling in a wide variety of biological mechanisms, and its dysfunction has been highly related to with favorable tumor microenvironment, cancer progression, and chemotherapy resistance [40]. The PI3K-AKT pathway is a frequently altered signaling pathway in GIC, the aberrant activation of which is one of the most frequent events in human cancer and play an important part in regulating cell growth, differentiation, migration, and survival, as well as angiogenesis and metabolism [41]. There is growing evidence that MAPK signaling plays an significant role in various physiological processes, including cell growth, differentiation, and apoptotic cell death and abnormal activation of this pathway may contribute to the pathogenesis of various human cancer types including GIC [42]. These results revealed that miR-20a may be associated with these important biological processes during the initiation and progression of GIC.

To gain further insights into the function and mechanisms of miR-20a involved in GIC, construction of the PPI network with the target genes of miR-20a and the screening of crucial hub genes were carried out. These hub genes were predominantly involved in some key pathways, most of which have been validated to be involved in GIC. In addition, emerging evidence has supported the roles of Central carbon metabolism for monitoring disease progression and therapy response and is responsible for the impairment of vital homeostatic processes in dopaminergic cells including neurotransmitter mechanisms, axonal transport of vesicles and cell survival [43]. The microRNAs in cancer pathway indicated that miR-20a provides a central node in cancer occurrence and development [44]. Emerging evidence has identified FoxO transcription factors to be the central regulators for cellular homeostasis, playing an important role during a large number of cellular activities ranging from development, cell signaling, and cancer initiation to cell metabolism [45]. The hub genes which were identified in the PPI network analysis could play a significant part in the aberrant signaling pathways and may provide potential targets for future research.

Subsequently, according to module analysis, significant modules were identified. To explore the biological activities of the genes involved in these modules, we then conducted KEGG enrichment analysis. The analysis results revealed that the module nodes were particularly enriched in a series of significant signaling pathways. Most of the enriched pathways were highly associated with occurrence and development of GIC based on PubMed literature reports mentioned above. In addition, Ubiquitin mediated proteolysis is responsible for regulating various cellular processes, and abnormal activation of these enzymes may lead to the pathogenesis of human diseases [46]. The spliceosome has been identified as a large protein complex for guiding pre-mRNA splicing in eukaryotic cells and the abnormal expression of it may lead to carcinogenesis [47]. Endocytosis has been regarded as a long-term mechanism of active transport as elected extracellular molecules are engulfed into intracellular spaces with energy consumption and thus has a great role in every aspects of tumor initiation and progression [48]. VEGF signaling has now been recognized as one of the most important regulatory factors in stimulating endothelial cells to promote both developmental and pathological angiogenesis [49]. It has been confirmed that VEGF is significantly involved in the initiation, progression, and recurrence of tumors, and may provide therapeutic target for colorectal cancer [50]. Previous evidence has indicated that HIF-1 signaling provides a central node to cancer dormancy and cancer metabolism [51]. Meanwhile, emerging evidence has supported that activation of HIF-1 signaling is significantly correlated with increasing stemness activity and causing cancer initiation and progression [52]. Studies have convinced the roles of Ras signaling in various types of cancers, and targeting RAS signaling may provide a potential therapeutic target in the treatment of colorectal cancer [53]. These results also revealed the potential mechanism of miR-20a involved in GIC again.

Recently, many studies on function and mechanism of miR-20a have been published [54]. Emerging evidence has supported the roles of miR-20a in regulating apoptotic genes that are related to TNF-related apoptosis-inducing ligand sensitivity of CRC [55]. As a result, targeting miR-20a may provide a promising method to promote apoptosis. Moreover, previous studies have revealed that miR-20a could induce epithelial-mesenchymal transition (EMT) by regulating Smad4 and TIMP2 expression and promote CRC invasion and metastasis by regulating GABBR1 [29, 56]. Meanwhile, there is growing evidence that miR-20a plays a significant role in inducing CRC cell senescence through targeting SENP1, and then promoted the invasiveness of CRC cells [57]. These studies together with the findings from our bioinformatic analysis may provide help for understanding the function and mechanism of miR-20a involved in GIC. They should be further confirmed through molecular biological experiments.

There are some limitations in the present study. Firstly, though we have performed a thorough search for screening associated literatures, the number of enrolled studies was still relatively small and limited ethnicities were evaluated. Secondly, potential publication bias was detected in the present study, which may overshadow our promising conclusions. Thirdly, because of insufficient data, we failed to investigate the potential for confounding by other demographic and clinical factors. In addition, the results of the present study were solely based on meta-analysis and bioinformatics, which were not verified by in vitro or in vivo experiments. Regardless of that, by using comprehensive meta-analysis and several integrated bioinformatics technologies, we not only validated the biomarker performance of miR-20a in predicting the survival outcomes of GIC, but preliminarily explored the potential underlying mechanisms.

## Conclusions

In summary, the present study demonstrated that overexpression of miR-20a is associated with poor prognosis of patients in GIC and may function as a useful prognostic indicator and a promising therapeutic target in GIC. The identified critical hub proteins and signaling pathways by integrative bioinformatic analysis may help improve the understanding of the underlying molecular mechanisms of miR-20a in the occurrence and progression of GIC, and additionally serve as candidate biomarkers and potential therapy targets in GIC. Nevertheless, more experiments with larger sample sizes should be conducted for further confirming the present results.

## Data Availability

The data supporting the conclusions of this article is within the article.

## Abbreviations

GIC: Gastrointestinal cancer
CRC: Colorectal cancer
GC: Gastric cancer
HR: Hazard ratio
CI: Confidence interval
NOS: Newcastle-Ottawa Scale
GO: Gene Ontology
KEGG: Kyoto Encyclopedia of Genes and Genomes
PPI: protein–protein interaction
DAVID: Database for Annotation, Visualization, and Integrated Discovery
BP: Biological processes
CC: Cell component
MF: Molecular function
